# Increases in inflammatory and CD14^dim^/CD16^pos^/CD45^pos^ patrolling monocytes in sepsis: correlation with final outcome

**DOI:** 10.1186/s13054-018-1977-1

**Published:** 2018-03-03

**Authors:** Gabriela Gainaru, Antonios Papadopoulos, Iraklis Tsangaris, Malvina Lada, Evangelos J. Giamarellos-Bourboulis, Aikaterini Pistiki

**Affiliations:** 1 4th Department of Internal Medicine, National and Kapodistrian University of Athens, Medical School, ATTIKON University hospital, 1 Rimini Street, 12462 Athens, Greece; 2 2nd Department of Critical Care Medicine, National and Kapodistrian University of Athens, Medical School, Athens, Greece; 3 2nd Department of Internal Medicine, Sismanogleion Athens Hospital, Athens, Greece

**Keywords:** Sepsis, Survival, Inflammatory monocytes, Patrolling monocytes, Gram-negative

## Abstract

**Background:**

Evidence on the changes in the absolute counts of monocyte subpopulations in sepsis is missing.

**Methods:**

Firstly, absolute counts of circulating CD14^pos^/HLA-DR^pos^/CD45^pos^ monocytes were measured by flow cytometry in 70 patients with Gram-negative sepsis and in 10 healthy volunteers. In the second phase, immunophenotyping was performed and the absolute count of circulating inflammatory monocytes and of circulating CD14^dim^/CD16^pos^/CD45^pos^ patrolling monocytes were measured in another 55 patients and 10 healthy volunteers. Measurements were repeated on days 3, 7, and 10. Results were correlated with survival after 28 days.

**Results:**

Greater numbers of CD14^pos^/HLA-DR^pos^/CD45^pos^ monocytes were found on day 1 in survivors compared to nonsurvivors (*p* = 0.030). Receiver operating characteristic (ROC) analysis showed that a cutoff higher than 337 cells/mm^3^ on day 1 could discriminate between survivors and nonsurvivors with a positive predictive value (PPV) of 91.1%. Logistic regression including Sequential Organ Failure Assessment (SOFA) score and Acute Physiology and Chronic Health Evaluation (APACHE) II score showed that an absolute count greater than 337 cells/mm^3^ was independently associated with unfavorable outcome (odds ratio (OR) 0.19, *p* = 0.050). The absolute counts of inflammatory and of CD14^dim^/CD16^pos^/CD45^pos^ monocytes were greater in patients than healthy controls during the entire 10 days of follow-up. The absolute counts on day 3 of CD14^dim^/CD16^pos^/CD45^pos^ monocytes were greater in survivors than nonsurvivors (*p* = 0.027). ROC analysis revealed that the cutoff at 27 cells/mm^3^ could discriminate between survivors and nonsurvivors with PPV of 94.1%. Logistic regression including age, SOFA score, and APACHE II score showed that an absolute count greater than 27 cells/mm^3^ was independently associated with unfavorable outcome (OR 0.06, *p* = 0.033). Logistic regression analysis showed that intra-abdominal infection on day 1 was predictive of low CD14^dim^/ CD16^pos^/CD45^pos^ count on day 3.

**Conclusion:**

Circulating counts of inflammatory and patrolling monocytes are greatly increased in Gram-negative sepsis. Absolute counts of CD14^pos^/HLA-DR^pos^/CD45^pos^ monocytes on day 1 and CD14^dim^/CD16^pos^/CD45^pos^ monocytes on day 3 are independently associated with final outcome.

**Trial registration:**

ClinicalTrials.gov, NCT01223690. Registered retrospectively on 18 October 2010.

**Electronic supplementary material:**

The online version of this article (10.1186/s13054-018-1977-1) contains supplementary material, which is available to authorized users.

## Background

Immune status in sepsis varies during the course of the disease. The early stages are characterized by a phase of excessive inflammation also known as the “cytokine storm”. In this stage, blood monocytes are overfunctioning for the production of cytokines. As the syndrome progresses, the proinflammation stage is followed by a phase of immune-suppression that is characterized by the inability for cytokine secretion [1]. In this stage monocytes are functionally deactivated, and this is shown by the decreased expression of the human leukocyte antigen (HLA) class II on their cell surface [2]. However, it is also possible that these two phases of proinflammation and anti-inflammation may occur simultaneously. Monocytes can be classified into three phenotypical and functionally distinct subpopulations based on the expression of the lipopolysaccharide receptor CD14 and of the FcγIII receptor CD16 on their cell membranes. According to this classification, monocytes are divided into the “classical or inflammatory monocytes” characterized by a bright expression of CD14 and lack of expression of CD16, the “intermediate monocytes” that express both receptors; and the “nonclassical” monocytes with patrolling function that express predominantly CD16 [3, 4]. Data on the contribution of patrolling monocytes to the pathogenesis of sepsis are still missing. Regarding the role of classical monocytes, available data are mainly focused on the change in their percentage whereas data on their absolute counts are missing [5, 6].

The aim of the present study was to investigate the changes in the absolute counts of monocyte subpopulations in sepsis. For reasons of study homogeneity, the study was limited to patients with infections either proven or highly suspected to be caused by Gram-negative bacteria.

## Methods

### Study design

This is a nested observational clinical study that was approved by the Ethics Committee of the ATTIKON University hospital (266/27-07-09). Written informed consent was provided by patients or by their legal representatives for patients unable to consent. This study was approved as a substudy of a randomized clinical study investigating the role of intravenous clarithromycin for the management of patients with proven or suspected Gram-negative sepsis [7]. The study is registered at https://clinicaltrials.gov/ct2/show/NCT01223690 (NCT01223690).

The study design comprised two phases. In the first phase, we investigated how early counts of CD14^pos^/HLA-DR^pos^/CD45^pos^ monocytes are associated with final outcome. If analysis of this phase yielded significant results, the study was allowed to proceed to the second phase. In the second phase, immunophenotyping of monocytes subpopulations was performed and absolute counts of inflammatory and patrolling monocytes were determined. The inclusion criteria for both phases were: 1) age more than or equal to 18 years; 2) severe sepsis or septic shock as defined by the Sepsis-2 definitions that were applying at that time [8]; 3) blood sampling blood within 24 h from the first organ failure; and 4) presence of acute pyelonephritis, intra-abdominal infection, and primary Gram-negative bacteremia. Patients with primary Gram-negative bacteremia were enrolled provided that within the time frame of 24 h since the first organ failure information was given by the microbiology laboratory on the growth of a Gram-negative species in blood culture.

Exclusion criteria were: 1) infection by the human immunodeficiency virus (HIV)-1; 2) neutropenia defined as less than 1000 neutrophils/mm^3^; 3) intake of chemotherapy; and 4) chronic corticosteroid intake defined as more than 0.4 mg/kg equivalent prednisone daily for more than 15 consecutive days.

Severe sepsis was defined as infection accompanied by at least two signs of the systemic inflammatory response syndrome (SIRS) and aggravated by at least one organ dysfunction. Septic shock was defined as severe sepsis aggravated with systolic blood pressure below 90 mmHg necessitating the administration of vasopressors despite adequate fluid resuscitation [8]. Acute pyelonephritis was defined as the presence of at least two of the following: 1) dysuria and pain on palpation of the left or right costovertebral angle; 2) more than 10 white blood cells/high-power field (hpf) of centrifuged urine; and 3) compatible findings on renal ultrasound [8]. An intra-abdominal infection was defined as the presence of both clinical signs and radiological findings on abdominal ultrasound or computed tomography compatible with an intra-abdominal infection [8]. Demographics, severity scores, white blood cell count, biochemistry, blood gases, type of infection, microbiology, comorbidities, and type of organ dysfunction were recorded. Survival was also recorded for 28 days.

Twenty healthy volunteers well-matched to sepsis patients for age and gender were also studied; 10 were studied at each phase of the study. At least one volunteer sample was running on the same day as patient samples.

From each patient and healthy volunteer, 2 ml whole blood were collected in one EDTA tube (Becton Dickinson, Cockeysville, MD, USA) under sterile conditions after venipuncture of one forearm vein at four time intervals: during the first 24 h from the first organ failure (day 1), two days later (day 3), 6 days later (day 7), and 9 days later (day 10).

### Laboratory investigation

Whole blood was incubated with the following combinations of monoclonal antibodies: a) anti-CD14 at the fluorochrome fluorescein isothiocyanate (FITC, emission 525 nm, clone RMO25, Immunotech, Marseille, France), anti-HLA-DR-DP-DQ at the fluorochrome RD1 (phycoerythrin (PE), emission 575 nm, clone 9-49 I3, CYTO-STAT, Miami, Florida, USA), and anti-CD45 at the fluorochrome phycoerythrin-cyanine 5 (PC5, emission 650 nm, clone J.33, Immunotech); and b) anti-CD14 at the fluorochrome FITC (clone RMO25, Immunotech), anti-CD16 at the fluorochrome PE (clone 3G8, Immunotech), and anti-CD45 at the fluorochrome PC5 (clone J.33, Immunotech). Red blood cells were lysed using VersaLyse Lysing Solution (Beckman Coulter, Immunotech, Marseille, France). White blood cells were analyzed after running through the Cytomics FC-500 flow cytometer with gating for monocytes based on their characteristic SS/CD45 expression. Fluorospheres (Immunotech) were used for the determination of absolute counts. Cells stained with IgG isotypic controls at the fluorochromes FITC and PE (clone 679.1Mc7, Immunotech) were running in parallel so that background staining could be subtracted. The technicians were blind to the clinical data of the patients.

### Power of the study

We hypothesized that after receiver operator characteristic (ROC) curve analysis of the absolute counts of CD14^pos^/HLA-DR^pos^/CD45^pos^ a cutoff associated with positive predictive value (PPV) more than 90% for survival could be found. To achieve this, with power more than 80% at the 10% level of significance, we calculated that at least 60 patients should be enrolled at the first phase. To adjust for patients dying prior to day 3, 70 patients were studied at the first phase. It was predefined that if analysis was positive at the end of the first phase, the study would proceed to the second phase with a similar number of patients.

### Statistical analysis

The absolute cell counts are expressed as medians and 95% confidence intervals (CIs) or interquartile range (IQR) and compared by the Mann-Whitney *U* test. Comparisons of baseline demographics between patients enrolled in each phase of the study and between survivors and nonsurvivors were performed by the Fisher exact test for qualitative variables and by the Student’s test for quantitative variables. ROC curve analysis was also performed to identify a cutoff for the absolute count of CD14^pos^/HLA-DR^pos^/CD45^pos^ on day 1 and of CD14^dim^/CD16^pos^/CD45^pos^ patrolling monocytes on day 3 that could discriminate between survivors and nonsurvivors with PPV more than 90%. Odds ratio (ORs) and 95% CIs for unfavorable outcome based on the cutoffs were determined according to Mantel-Haenszel’s statistics. Comparative survival analysis was performed by the log-rank test. Comparisons of baseline characteristics between patients above or below these cutoffs were performed as described above. Variables with a *p* value lower than 0.050 entered the equations of step-wise forward logistic regression analysis as independent variables; the cutoffs of monocytes or 28-day mortality were the dependent variable. ORs and 95% CIs were calculated. Any value of *p* below 0.05 was considered statistically significant.

## Results

**Fig. 1:**
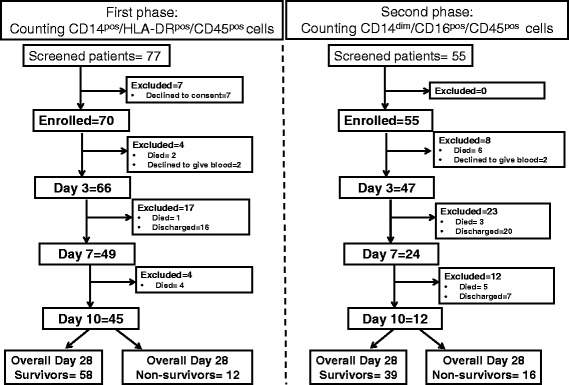
Study flow during the two phases

The study flowchart is shown in Fig. 1. The study was divided into two phases. In the first phase, absolute counts of CD14^pos^/HLA-DR^pos^/CD45^pos^ monocytes were determined in 70 patients and 10 healthy volunteers. Demographic characteristics of patients participating in both study phases are shown in Additional file 1 (Table S1).

Absolute cell counts of CD14^pos^/HLA-DR^pos^/CD45^pos^ monocytes on days 1 and 3 did not differ between patients allocated to the two groups of treatment (Additional file 2: Figure S1A). Since all-cause mortality was similar in the two groups [7], data from both groups were pooled and analyzed together.

**Fig. 2:**
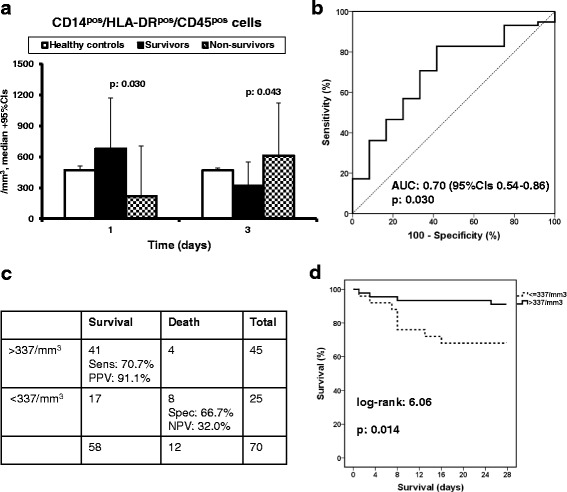
Association between final outcome and absolute counts of circulating CD14^pos^/HLA-DR^pos^/CD45^pos^ monocytes. **a** Absolute counts of CD14^pos^/HLA-DR^pos^/CD45^pos^ monocytes of survivors and nonsurvivors on days 1 and 3 and healthy volunteers. *P* values refer to comparisons between survivors and nonsurvivors. **b** ROC curve of the absolute count of CD14^pos^/HLA-DR^pos^/CD45^pos^ monocytes on day 1 to predict survival after 28 days. **c** Predictive value of an absolute count of CD14^pos^/HLA-DR^pos^/CD45^pos^ monocytes more than 337 cells/mm^3^ on day 1 for survival after 28 days. **d**) Comparative survival in relation to the absolute count of CD14^pos^/HLA-DR^pos^/CD45^pos^ monocytes on day 1. AUC area under curve, CI confidence interval, NPV negative predictive value, PPV positive predictive value, Sens sensitivity, Spec specificity

Absolute cell counts of CD14^pos^/HLA-DR^pos^/CD45^pos^ monocytes on day 1 were significantly higher in survivors compared to nonsurvivors (Fig. 2a). Based on this observation, a ROC curve was designed and a cutoff value of 337 cells/mm^3^ could discriminate between survivors and nonsurvivors with PPV of 91.1% (Fig. 2b, c). These results were further confirmed after survival analysis showing prolonged survival among patients with more than 337 cells/mm^3^ on day 1 (Fig. 2d). The OR for unfavorable outcome among patients with more than 337 cells/mm^3^ was 0.21 (95% CI 0.06–0.78; *p* = 0.020). Median (IQR) count of CD14^pos^/HLA-DR^pos^/CD45^pos^ monocytes on day 1 was 533.7 (846.1)/mm^3^ among patients without acute kidney injury (AKI) and 852.7 (1181.9)/mm^3^ among patients with AKI (*p* = 0.275). Among patients remaining in phase 1 of the study after day 3, median (IQR) count of CD14^pos^/HLA-DR^pos^/CD45^pos^ monocytes on day 7 was 192 (494.8)/mm^3^ among survivors and 321.9 (365.3)/mm^3^ among nonsurvivors (*p* = 0.620); the respective values on day 10 were 900.5 (704.8)/mm^3^ and 254.4 (820.3)/mm^3^ (*p* = 0.312).

These results generated the question whether the defined cutoff for CD14^pos^/HLA-DR^pos^/CD45^pos^ monocytes on day 1 is an independent predictor of final outcome. As shown in Additional file 3 (Table S2), the only variables that differed significantly between nonsurvivors and survivors at baseline were Acute Physiology and Chronic Health Evaluation (APACHE) II and Sequential Organ Failure Assessment (SOFA) scores. In the logistic regression analysis, APACHE II score, SOFA score, and an absolute CD14^pos^/HLA-DR^pos^/CD45^pos^ monocyte count on day 1 greater than 337 cells/mm^3^ were entered as independent variables; 28-day outcome was the dependent variable. Analysis showed that an absolute CD14^pos^/HLA-DR^pos^/CD45^pos^ monocyte count on day 1 greater than 337 cells/mm^3^ was independently associated with favorable 28-day outcome (Table 1).

**Table 1:** Logistic regression analysis of variables associated with unfavorable outcome in each phase of the study

	Odds ratio	95% confidence interval	*p* value
Phase 1			
SOFA score on day 1	1.60	1.15–2.22	0.005
APACHE II score on day 1	0.98	0.86–1.13	0.825
CD14^pos^/HLA-DR^pos^/CD45^pos^ > 337/mm^3^ on day 1	0.19	0.04–1.00	0.050
Phase 2			
Age	1.07	0.98–1.17	0.105
SOFA score on day 1	1.73	0.99–3.00	0.050
APACHE II score on day 1	1.05	0.91–1.21	0.526
CD14^dim^/CD16^bri^/CD45^pos^ > 27/mm^3^ on day 3	0.06	0.00–0.79	0.033

*APACHE* Acute Physiology and Chronic Health Evaluation, *SOFA* Sequential Organ Failure Assessment

**Fig. 3:**
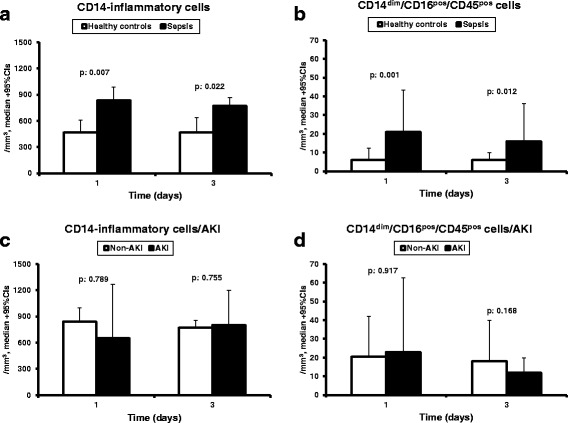
Absolute counts of monocyte subpopulations in sepsis. **a** Absolute counts of inflammatory monocytes and of **b** CD14^dim^/CD16^pos^/CD45^pos^ patrolling monocytes of healthy controls and of patients with sepsis on days 1 and 3. **c** Absolute counts of inflammatory monocytes and of **d** CD14^dim^/CD16^pos^/CD45^pos^ patrolling monocytes of patients with sepsis on days 1 and 3 divided into those without and those with acute kidney injury (AKI). *P* values refer to the indicated comparisons

Since the impact of the absolute count of CD14^pos^/HLA-DR^pos^/CD45^pos^ monocytes on survival was confirmed after the first phase, we proceeded with the second phase where absolute counts of subpopulations of monocytes were measured in 55 patients and 10 healthy volunteers (Fig. 1). Three monocyte subpopulations were defined: CD14^pos^/CD16^neg^/CD45^pos^ and CD14^pos^/CD16^pos^/CD45^pos^ monocytes analyzed and reported together as inflammatory monocytes, and CD14^dim^/CD16^pos^/CD45^pos^ reported as patrolling monocytes. The absolute counts of both inflammatory and patrolling monocytes were significantly greater among patients than healthy controls (Fig. 3a, b); no differences were found between patients without and with AKI (Fig. 3c, d). Among patients remaining in phase 2 of the study after day 3, median (IQR) counts of inflammatory monocytes was 641 (592.3)/mm^3^ on day 7 (*p* = 0.204 vs controls) and 669 (519)/mm^3^ on day 10 (*p* = 0.049 vs controls). Those of CD14^dim^/CD16^pos^/CD45^pos^ patrolling monocytes were 14 (20)/mm^3^ on day 7 (*p* = 0.043 vs controls) and 25.5 (28.5)/mm^3^ on day 10 (*p* = 0.011 vs controls).

As a next step, we explored if the described changes in the subpopulations of monocytes are associated with final outcome. The absolute cell counts on days 1 and 3 did not differ between patients allocated to placebo or clarithromycin treatment (Additional file 2: Figure S1B and C). Since all-cause mortality was similar in the two groups [7], data from both groups were pooled and analyzed together.

**Fig. 4:**
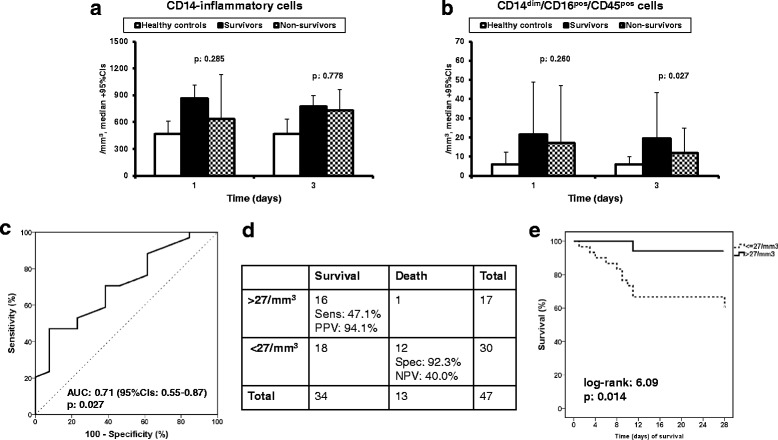
Association between final outcome and absolute counts of circulating CD14^dim^/CD16^pos^/CD45^pos^ patrolling monocytes on day 3. **a** Absolute counts of inflammatory monocytes of survivors and nonsurvivors on days 1 and 3 and healthy volunteers. *P* values refer to comparisons between survivors and nonsurvivors. **b** Absolute counts of CD14^dim^/CD16^pos^/CD45^pos^ patrolling monocytes of survivors and nonsurvivors on days 1 and 3 and healthy volunteers. *P* values refer to comparisons between survivors and nonsurvivors. **c** ROC curve of the absolute count of patrolling monocytes on day 3 to predict survival after 28 days. **d** Predictive value of an absolute count of patrolling monocytes greater than 27 cells/mm^3^ on day 3 for survival after 28 days. **e** Comparative survival for patients according to the number of patrolling monocytes on day 3. AUC area under curve, CI confidence interval, NPV negative predictive value, PPV positive predictive value, Sens sensitivity, Spec specificity

No differences in cell counts of inflammatory monocytes were observed between survivors and nonsurvivors for any day of sampling (Fig. 4a). Regarding CD14^dim^/CD16^pos^/CD45^pos^ patrolling monocytes, significant differences between survivors and nonsurvivors were found on day 3 (Fig. 4b). ROC analysis revealed that a cutoff value of 27 cells/mm^3^ could discriminate between survivors and nonsurvivors with PPV 94.1% (Fig. 4c, d). This finding was further confirmed after survival analysis (Fig. 4e). The OR for unfavorable outcome among patients with more than 27 cells/mm^3^ was 0.09 (95% CI 0.01–0.80; *p* = 0.031).

These results generated the question whether the defined cutoff for CD14^dim^/CD16^pos^/CD45^pos^ patrolling monocytes on day 3 is an independent predictor of final outcome. As shown in Additional file 4 (Table S3), the only variables that differed significantly between nonsurvivors and survivors at baseline were age, APACHE II score, and SOFA score. In the logistic regression analysis, age, APACHE II score, SOFA score, and an absolute count of CD14^dim^/CD16^pos^/CD45^pos^ patrolling monocytes on day 3 greater than 27 cells/mm^3^ were entered as independent variables; 28-day outcome was the dependent variable. Analysis showed that an absolute count of CD14^dim^/CD16^pos^/CD45^pos^ patrolling monocytes on day 3 greater than 27 cells/mm^3^ was independently associated with favorable 28-day outcome (Table 1).

Next, we asked the question whether clinical signs at admission could predict low counts of patrolling monocytes on day 3. The frequencies of acute pyelonephritis and intra-abdominal infection were significantly different between patients with ≤ 27 cells/mm^3^ or greater than 27 cells/mm^3^ CD14^dim^/CD16^pos^/CD45^pos^ patrolling monocytes (Table 2). These two clinical characteristics were entered into the logistic regression analysis as independent variables. Analysis revealed that the presence of an intra-abdominal infection was the only variable associated with low CD14^dim^/CD16^pos^/CD45^pos^ patrolling monocyte counts on day 3 (Table 3).

**Table 2:** Differences in baseline characteristics of patients in relation to the absolute number of CD14^dim^/CD16^bri^/CD45^pos^ monocytes on day 3

	> 27 cells/mm^3^ (*n* = 17)	≤ 27 cells/mm^3^ (*n* = 30)	*p*
Male gender	6 (35.3%)	14 (46.7%)	0.546
Age (years)	69.5 ± 15.9	71.8 ± 17.4	0.658
APACHE II score	11.0 ± 8.2	13.3 ± 8.1	0.378
SOFA score	2.4 ± 2.1	3.2 ± 2.5	0.266
White blood cells (/mm^3^)	13,262.9 ± 6016.2	12,728.5 ± 4211.1	0.750
pO_2_/FiO_2_	326.7 ± 109.1	209.9 ± 129.3	0.094
C-reactive protein (mg/l)	136.4 ± 99.3	121.3 ± 118.1	0.679
Type of infection			
Acute pyelonephritis	13 (76.5%)	9 (30.0%)	0.003
Primary Gram-negative bacteremia	3 (17.6%)	4 (13.3%)	0.692
Acute intra-abdominal infection	1 (5.9%)	17 (57.6%)	0.001
Presence of at least one chronic disorder^a^	8 (47.1%)	15 (50.0%)	0.758
Presence of multiple organ failure	5 (35.7%)	7 (31.7%)	1.00

Values are shown as mean ± SD or *n* (%)

APACHE Acute Physiology and Chronic Health Evaluation, FiO2 fractional inspired oxygen, pO2 arterial oxygen tension, SOFA Sequential Organ Failure Assessment

^a^ Type 2 diabetes mellitus, chronic obstructive pulmonary disorder, chronic heart failure, chronic renal disease, solid tumor malignancy

**Table 3:** Step-wise forward logistic regression analysis of admission characteristics on day 1 to predict the chance for the presence of low circulating patrolling monocyte cell counts on day 3

	Odds ratio	95% confidence interval	*p* value
Acute pyelonephritis	0.52	0.09–2.90	0.453
Acute intra-abdominal infection	12.75	1.03–2.90	0.047

## Discussion

Circulating counts of inflammatory and patrolling monocytes are significantly increased up to at least 10 days in the course of Gram-negative sepsis. Exploratory analysis also identified an association between circulating CD14^dim^/CD16^pos^/CD45^pos^ patrolling monocytes on the third day of follow-up and 28-day outcome; the greater number of this subpopulation, the lower is the risk for unfavorable outcome. The presence of acute intra-abdominal infection on admission is a predictive sign for low circulating patrolling monocytes counts on day 3. It was also found that more than 337 CD14^pos^/HLA-DR^pos^/CD45^pos^ monocytes/mm^3^ on admission is associated with favorable outcome.

HLA-DR is expressed on activated CD14 monocytes that are capable of antigen presentation and perpetuation of the inflammatory reaction. In sepsis, the expression of HLA-DR on circulating CD14 monocytes and the number of HLA-DR molecules (mHLA-DR) on the monocyte surface are decreased and this is interpreted as an index of sepsis-induced immunosuppression [9, 10]. To date, decreased mHLA-DR is considered the gold standard for the identification of immunosuppression [11]. However, several problems seem to apply for the universal application of mHLA-DR as a diagnostic tool, which are mainly associated with the inter-laboratory variability of the assay.

Recent studies enrolling between 79 and 93 patients [12–14] (i.e., close to the enrolment of the present study) showed that the decrease in HLA-DR expression is greater in septic shock and among nonsurvivors as early as the first day of sampling. The expression of HLA-DR on CD14 monocytes was further deepening upon worsening on days 3 and 7 of follow-up. However, in these studies the percentage of CD14 cells expressing HLA-DR and mHLA-DR were studied and the absolute count of circulating CD14^pos^/HLA-DR^pos^/CD45^pos^ cells was not reported as in our study. This may explain why the absolute count of circulating CD14^pos^/HLA-DR^pos^/CD45^pos^ cells was different between survivors and nonsurvivors only on day 1 and not on the next days of follow-up. It may also be the case that the provided association of the absolute count of circulating CD14^pos^/HLA-DR^pos^/CD45^pos^ cells on day 1 is a reflection of the pathobiology of the host but that it cannot serve as a biomarker for the follow-up of the patient.

An expansion of CD16-expressing monocytes usually occurs in sepsis with a simultaneous reduction in inflammatory monocytes [5]. In two previous studies, one in sepsis and another in acute pancreatitis, the absolute counts of cells highly expressing both CD14 and CD16 were measured [15, 16]; however, the counts of patrolling monocytes were not reported.

At a functional level, patrolling monocytes are poorly phagocytic, have high antigen-presenting capacity, and express higher levels of Toll-like receptors (TLRs) compared with the other CD14 monocyte subpopulations. In addition, they bear a proinflammatory function by producing high levels of tumor necrosis factor (TNF)α and interleukin (IL)-1β but not IL-10 upon stimulation with lipopolysaccharide (LPS) [5, 17]. In a recent publication from our group we found that failure of adequate production of proinflammatory cytokines by circulating mononuclear cells 48 h after admission for sepsis was interpreted as sustaining the immunosuppression and it was associated with poor outcome. This defective production continued for at least 10 days [18]. Since CD14^dim^/CD16^pos^/CD45^pos^ cells are the monocyte subpopulation providing most of the TNFα production, the low cell count of patrolling monocytes on the third day in nonsurvivors could be associated with the immunosuppression of sepsis.

The current study is powered to define the absolute count of CD14^pos^/HLA-DR^pos^/CD45^pos^ on day 1 that impact on final outcome. Although the number of patients enrolled during the second phase of the study is rather small to provide definitive associations with final outcome, it clearly reveals a main pathobiological feature of Gram-negative sepsis—i.e., the increase in the studied monocyte subpopulations. However, the independent associations of the proposed cutoffs of circulating CD14^pos^/HLA-DR^pos^/CD45^pos^ cells on day 1 and of the circulating CD14^dim^/CD16^pos^/CD45^pos^ cells on day 3 with the final outcome proved through logistic regression analysis are remarkable.

## Conclusions

This study shows that circulating counts of inflammatory and patrolling monocytes are greatly increased in Gram-negative sepsis. Low absolute counts of CD14^dim^/CD16^pos^/CD45^pos^ patrolling monocytes on day 3 are associated with unfavorable outcome. The presence of acute intra-abdominal infection at baseline can predict the low absolute count of patrolling monocytes on day 3. In addition, an association with unfavorable outcome is also provided by low absolute counts of CD14^pos^/HLA-DR^pos^/CD45^pos^ on day 1. The present findings cannot support the use of the defined cutoffs of CD14^pos^/HLA-DR^pos^/CD45^pos^ and of CD14^dim^/CD16^pos^/CD45^pos^ cells as biomarkers for sepsis since validation by another independent cohort is warranted. However, they strongly provide evidence for the independent role of CD14^pos^/HLA-DR^pos^/CD45^pos^ cells at baseline and of the CD14^dim^/CD16^pos^/CD45^pos^ cells during progression of sepsis as determinants of the outcome of the septic host.

## Additional files

Additional file 1: **Table S1.** Demographic characteristics of patients in both study phases. (DOCX 16 kb)

Additional file 2: **Figure S1.** Absolute counts of circulating CD14^pos^/HLA-DR^pos^/CD45^pos^ monocytes and subpopulations of monocytes in relation to treatment allocation. Absolute counts of (A) CD14^pos^/HLA-DR^pos^/CD45^pos^ monocytes, (B) inflammatory monocytes, and (C) CD14^dim^/CD16^pos^/CD45^pos^ patrolling monocytes on days 1 and 3 between patients allocated to treatment with placebo and patients allocated to treatment with clarithromycin. *P* values refer to the indicated comparisons. (DOCX 105 kb)

Additional file 3: **Table S2.** Differences of baseline characteristics of day 1 between survivors and nonsurvivors of the first phase of the study. (DOCX 18 kb)

Additional file 4: **Table S3.** Differences of baseline characteristics of day 1 between survivors and nonsurvivors of the second phase of the study. (DOCX 18 kb)

## Abbreviations

AKI: Acute kidney injury
APACHE: Acute Physiology and Chronic Health Evaluation
CD: Cluster of differentiation
CI: Confidence interval
FcγIII: Fragment crystallizable-γ III
FITC: Fluorescein isothiocyanate
HLA-DR: Human leukocyte antigen-D related
hpf: High-power field
IL: Interleukin
IQR: Interquartile range
LPS: Lipopolysaccharide
OR: Odds ratio
PC5: Phycoerythrin-cyanine 5
PE: Phycoerythrin
PPV: Positive predictive value
ROC: Receiver operating characteristic
SD: Standard deviation
SOFA: Sequential Organ Failure Assessment
SS: Side scatter
TLR: Toll-like receptor
TNF: Tumor necrosis factor
