# Supplementary material for: Increases in inflammatory and CD14dim/CD16pos/CD45pos patrolling monocytes in sepsis: correlation with final outcome
Source: Crit Care. 2018 Mar 3;22:56. doi: 10.1186/s13054-018-1977-1 (PMC5834896; doi:10.1186/s13054-018-1977-1)
Supplement: Supplementary file 1 — Table S1. Demographic characteristics of patients in both study phases. (DOCX 16 kb) [file 13054_2018_1977_MOESM1_ESM.docx]

Additional file 1: **Table S1** Demographic characteristics of patients in both study phases

|  | **Phase 1 (n=70)** | | **Phase 2 (n= 55)** | **p** |
| --- | --- | --- | --- | --- |
| Sex (Male/Female) (n,%) | 34 (48.6) / 36 (51.4) | | 24 (43.6) / 31 (56.3) | 0.594 |
| Age (Years: mean ± SD) | 70.5 ±17.9 | | 71.7 ± 16.1 | 0.606 |
| APACHE II (mean ± SD) | 12.9 ± 7.1 | | 13.9 ± 9.0 | 0.512 |
| White blood cells (mean ±SD) | 14,255.9 ± 7,129.8 | | 13, 379.1 ± 5,657.6 | 0.463 |
| SOFA (mean ± SD) | | 5.2 ± 3.5 | 3.2 ± 2.7 | 0.002 |
| Death (n, %) | | 12 (17.1) | 16 (29.1) | 0.133 |
| **Infections** | |  |  |  |
| Acute intrabdominal infection (n, %) | | 24 (34.3) | 22 (40.0) | 0.577 |
| Acute pyelonephritis (n, %) | | 37 (52.9) | 26 (47.3) | 0.591 |
| Primary Gram-negative bacteremia (n, %) | | 9 (12.9) | 7 (12.7) | 0.180 |
| **Isolated pathogens** | |  |  |  |
| *Escherichia coli* (n, %) | | 16 (22.9) | 9 (16.4) | 0.395 |
| *Klebsiella pneumoniae* (n, %) | | 7 (10) | 9 (16.4) | 0.272 |
| *Pseudomonas aeruginosa* (n, %) | | 5 (7.1) | 3 (5.5) | 0.701 |
| Other Gram-negatives (n, %) | | 5 (7.1) | 8 (14.5) | 0.178 |

Abbreviations: APACHE: acute physiology and chronic health evaluation, SOFA: sequential organ failure assessment
