# Supplementary material for: Increases in inflammatory and CD14dim/CD16pos/CD45pos patrolling monocytes in sepsis: correlation with final outcome
Source: Crit Care. 2018 Mar 3;22:56. doi: 10.1186/s13054-018-1977-1 (PMC5834896; doi:10.1186/s13054-018-1977-1)
Supplement: Supplementary file 2 — Figure S1. Absolute counts of circulating CD14pos/HLA-DRpos/CD45pos monocytes and subpopulations of monocytes in relation to treatment allocation. Absolute counts of (A) CD14pos/HLA-DRpos/CD45pos monocytes, (B) inflammatory monocytes, and (C) CD14dim/CD16pos/CD45pos patrolling monocytes on days 1 and 3 between patients allocated to treatment with placebo and patients allocated to treatment with clarithromycin. P values refer to the indicated comparisons. (DOCX 105 kb) [file 13054_2018_1977_MOESM2_ESM.docx]

**
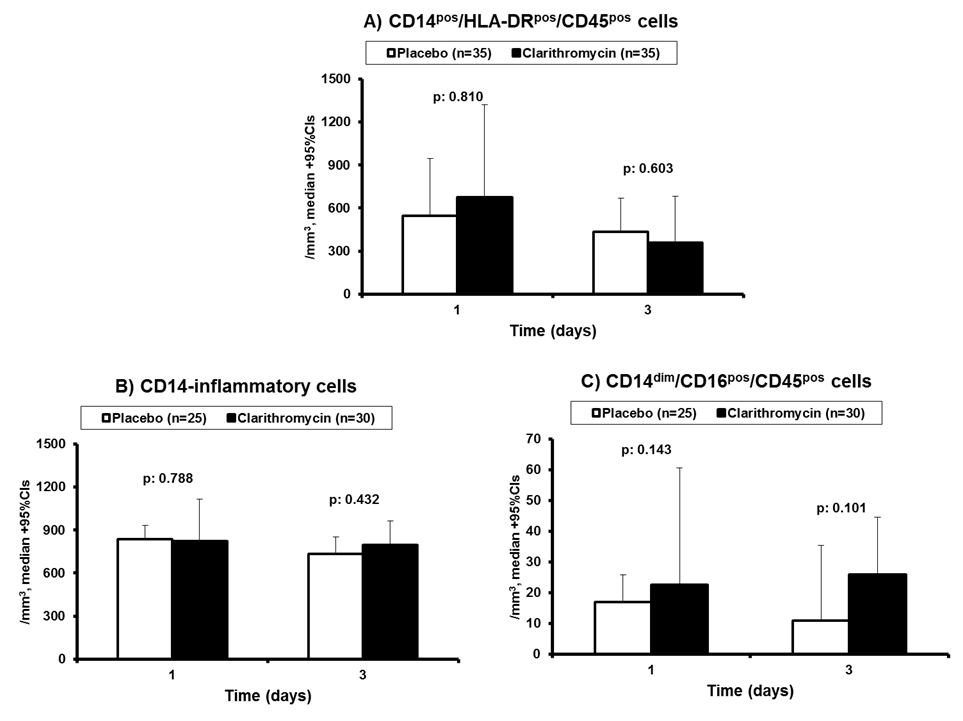
**

**Supplementary Figure 1 Absolute counts of circulating CD14^pos^/HLA-DR^pos^/CD45^pos^ monocytes and sub-populations of monocytes in relation to treatment allocation** Absolute counts of A) CD14^pos^/HLA-DR^pos^/CD45^pos^ monocytes; B) inflammatory monocytes; and C) CD14^dim^/CD16^pos^/CD45^pos^ patrolling monocytes on days 1 and 3 between patients allocated to treatment with placebo and patients allocated to treatment with clarithromycin. P values refer to the indicated comparisons.
