# Supplementary material for: Increases in inflammatory and CD14dim/CD16pos/CD45pos patrolling monocytes in sepsis: correlation with final outcome
Source: Crit Care. 2018 Mar 3;22:56. doi: 10.1186/s13054-018-1977-1 (PMC5834896; doi:10.1186/s13054-018-1977-1)
Supplement: Supplementary file 3 — Table S2. Differences of baseline characteristics of day 1 between survivors and nonsurvivors of the first phase of the study. (DOCX 18 kb) [file 13054_2018_1977_MOESM3_ESM.docx]

Additional file 3: **Table S2** Differences of baseline characteristics of day 1 between survivors and non-survivors of the first phase of the study.

|  | **Survivors (n=58)** | **Non-survivors (n=12)** | **p** |
| --- | --- | --- | --- |
| Male gender (n, %) | 29 (50.0) | 5 (41.7) | 0.754 |
| Age (years, mean ± SD) | 68.9 ± 18.9 | 75.6 ± 11.6 | 0.244 |
| APACHE II score (mean ± SD) | 11.9 ± 6.4 | 18.9 ± 8.5 | 0.006 |
| SOFA score (mean ± SD) | 4.32 ± 2.43 | 9.16 ± 5.00 | <0.0001 |
| White blood cells (/mm^3^, mean ± SD) | 14,209.7 ± 6,792.6 | 14,497.2 ± 8,928.4 | 0.906 |
| pO_2_/FiO_2_ (mmHg, mean ± SD) | 323.1 ± 95.8 | 349.9 ± 152.6 | 0.530 |
| C-reactive protein (mean ± SD, mg/l) | 122.3 ± 84.6 | 102.8 ± 182.6 | 0.567 |
| **Type of infection** |  |  |  |
| Acute pyelonephritis (n, %) | 34 (58.4) | 3 (25.0) | 0.055 |
| Primary Gram-negative bacteremia (n, %) | 6 (10.3) | 3 (25.0) | 0.177 |
| Acute intrabdominal Infection (n, %) | 18 (31.0) | 6 (50.0) | 0.316 |
| Presence of at least one chronic disorder (n, %)* | 32 (55.2) | 10 (83.3) | 0.106 |

*type 2 diabetes mellitus, chronic obstructive pulmonary disorder, chronic heart failure, chronic renal disease, solid tumor malignancy

Abbreviations APACHE: acute physiology and chronic health evaluation; SOFA: sequential organ failure assessment
