# Supplementary material for: Increases in inflammatory and CD14dim/CD16pos/CD45pos patrolling monocytes in sepsis: correlation with final outcome
Source: Crit Care. 2018 Mar 3;22:56. doi: 10.1186/s13054-018-1977-1 (PMC5834896; doi:10.1186/s13054-018-1977-1)
Supplement: Supplementary file 4 — Table S3. Differences of baseline characteristics of day 1 between survivors and nonsurvivors of the second phase of the study. (DOCX 18 kb) [file 13054_2018_1977_MOESM4_ESM.docx]

Additional file 4: **Table S3** Differences of baseline characteristics of day 1 between survivors and non-survivors of the second phase of the study.

|  | **Survivors (n=39)** | **Non-survivors (n=16)** | **p** |
| --- | --- | --- | --- |
| Male gender (n, %) | 16 (41.0) | 8 (50.0) | 0.565 |
| Age (years, mean ± SD) | 68.3 ± 16.7 | 80.3 ± 10.8 | 0.013 |
| APACHE II score (mean ± SD) | 11.6 ± 8.2 | 20.2 ± 8.3 | 0.001 |
| SOFA score (mean ± SD) | 2.61 ± 2.59 | 5.13 ± 2.25 | 0.001 |
| White blood cells (/mm^3^, mean ± SD) | 12,948.5 ± 5,736.9 | 14,578.6 ± 5,451.8 | 0.360 |
| pO_2_/FiO_2_ (mmHg, mean ± SD) | 324.9 ± 121.7 | 144.0 ± 73.2 | <0.0001 |
| C-reactive protein (mean ± SD, mg/l) | 108.3 ± 94.4 | 193.6 ± 158.6 | 0.028 |
| Type of infection |  |  |  |
| Acute pyelonephritis (n, %) | 20 (51.3) | 6 (37.5) | 0.389 |
| Primary Gram-negative bacteremia (n, %) | 3 (7.7) | 4 (25.0) | 0.175 |
| Acute intrabdominal Infection (n, %) | 16 (41.0) | 6 (37.5) | 1.00 |
| Presence of at least one chronic disorder (n, %)* | 21 (53.8) | 8 (50.0) | 1.00 |

*type 2 diabetes mellitus, chronic obstructive pulmonary disorder, chronic heart failure, chronic renal disease, solid tumor malignancy

Abbreviations APACHE: acute physiology and chronic health evaluation; SOFA: sequential organ failure assessment
